# Case of Hemorrhagic Disease, Following Venesection after Amputation, and Finally Terminating in Purpura

**Published:** 1833-03

**Authors:** Alex. Anderson

**Affiliations:** one of the Surgeons of the Western General Dispensary.


### HEMORRHAGIC DISEASE.

*Case of Hemorrhagic Disease, following Venesection after Amputation, and finally terminating in Purpura.* Read at the Harveian Society, on January the 21st, by ALEX. ANDERSON, Esq., one of the Surgeons of the Western General Dispensary.

JANUARY 31, 1831. Richard Crawford, aged thirty-nine, of phlegmatic temperament, was admitted a patient of the

Westminster General Dispensary, with disease of the right wrist, of six years' duration: this had originated from a sprain; inflammation supervened, and its consequences, suppuration, caries, and hectic fever, finally demanded the amputation of the wrist.

November 5, 1832. Amputation was performed half-way up the forearm. Three arteries being tied, the bleeding ceased. The stump was dressed in the usual mode. He bore the operation well, and without faintness. I was about to take my leave, when, upon his exerting himself somewhat, hemorrhage took place, and increased so much and so rapidly, as to render it imperative to search for the bleeding vessels: two were discovered, and secured, upon which the hemorrhagy ceased; but, after the lapse of a few minutes, there was an oozing, then a gush, from another vessel, which it was necessary to tie also. The stump was become much heated, and the action of its arteries remarkably violent. These, in connexion with another circumstance that occurred some weeks before, namely, an astonishing profusion of blood, which followed venesection on the back of the diseased hand, prepared me to expect further hemorrhage: it was, therefore, determined, if such should ensue, to repress it by the constant application of a cold solution of alum, to keep on the tourniquet, but slackened, as a ready help; to use no dressing besides a strap of adhesive plaster, and a pledget with spermaceti ointment, for the support of the wound; and to give full doses of the tincture of foxglove. Notwithstanding, a smart hemorrhage did take place next morning, and rather more than half a pint was lost before the tourniquet could be commanded. This seemed to have been induced by two causes: the getting out of bed, and a temporary excess of excitability of the heart and arteries; for, upon resuming his quiet posture in bed, and the abatement of the flush, the tourniquet was slackened, without any further hemorrhagy.

During five days there continued to be occasionally slight oozings of blood, which were always attendant on a flush and quickened circulation; and, but for the assiduous attention to rest, cold applications, and the use of the tincture of foxglove, would doubtless have become more frequent and greater in quantity.

The granulations were of a livid hue, and exceedingly disposed to bleed, up to the completion of the cicatrix, which was within three weeks from the day of the operation.

Upon examination of the diseased wrist, the cartilages of the joint had completely disappeared; the carpal bones separated and carious; the ligaments in some places absorbed, in others vastly thickened.

January 10th, 1833. Up to the present date, his health has continued to improve: he has become robust, works hard at the mangle, and is carrying out heavy loads of clothes; his wife being a laundress. Nothing worthy of remark occurred, except a patch of erythema near to the cicatrix, which came and was gone within the space of three days; and certainly nothing was further from my thoughts than that the hemorrhagic disposition was to be so soon and so awfully manifested as I am now about to relate.

January 11th. He had been seized the previous evening with vertigo, rigor, severe headach, and pains in the small of the back. His appearance is exactly that of a person in measles, on the first day of the eruption: there is tumidity of the face and neck; much coryza from the eyes and nostrils; cough; quick respiration; and a distinct eruption upon the extremities. He had had measles. The pulse was quiet and under ninety. Two opening pills and diaphoretic medicine (Liq. Ammon, Acet.) were prescribed.

January 12th, two o'clock. During the night he had vomited every thing he swallowed, and mixed with blood; and, since early this morning, had been purging every half-hour, indeed, more frequently, almost pure blood. The flush had changed to a lividity of the skin. The surface of the trunk and arms is thickly beset with spots of purpura, of the size of a split pea; some of them are pink or florid, others of a deep lead-colour or purple. On the face and lower extremities they are not so numerous. His wife first discovered the spots about nine this morning, on one side of the neck. The mucous membranes were quite as congestive as on yesterday, and, from the dark colour of the blood-vessels of the conjunctiva, the eye had a very death-like appearance, the gums were of a lead colour inclining to purple, the tongue covered with a thick milk-like coating, breathing very much oppressed, with much phlegm, which he expectorated easily. The vision, hearing, and touch were much impaired; not so his mental faculties, these he exercised perfectly when he heard what was said to him. The pulse varied much in rapidity and in strength, at times almost imperceptible, from 100 to 120. I bled him from the arm to the extent of eight ounces. After this operation he expressed himself relieved, he could distinguish objects more clearly, and could hear better. Upon inspiring deeply, he experienced no pain; the respiratory murmur distinctly audible. The actions of the heart feeble. Had passed little or no urine. He did not appear to suffer much from thirst, as a very little bit of orange satisfied him: indeed, I had sent for some oranges, intending that he should partake of their juice liberally, but this, like every thing else, was quickly vomited along with blood.

Ten P.M. He has been dead two hours; had passed no urine since last visit. The spots were less distinct, becoming diffused, and presenting the appearance of ecchymosis. The blood drawn at the last visit had a remarkable appearance. I had noticed how very quickly the crassamentum subsided from the serum, and I did expect to find buff on the surface; but there was no buff. The serum was jellied, resembling very weak size; the red crassamentum occupying the bottom of the vessel was fluid as molasses. The remedies prescribed were V.S., ad ℥viij. orange juice, and effervescing medicine with an excess of acid.

January 13, half-past two. The body was examined. The spots were become less distinct, all around them was extremely livid from ecchymosis. The neck tumid and livid. The right side of the chest flattened, and non-resonant. Upon opening the body, the blood was found to be fluid everywhere, and very dark coloured. The pleura of the right side was so adherent as not to admit of separation without the knife, throughout the whole extent of the pleura costalis; the subjacent lobe of lung was condensed; the other two lobes, with their investing pleura, were healthy. It may be remarked, that he had a very narrow escape fifteen years ago from a severe attack of pleurisy. The lungs of the left side healthy, strictly speaking, perhaps somewhat emphysematous. In the pleura of this side about half a pint of serum tinged with blood, whether from the diseased action, or from the division of some vessel by the knife, may be doubted. The lining of the air-tubes was much reddened, the heart quite healthy. The liquor pericardii perhaps superabundant. Spots of purpura, varying in number and in size from that of a pin's head to a split pea, were found throughout the whole mucous surface of the stomach and intestines, and upon the peritoneal coat of the termination of the ilium and commencement of the colon. The other viscera were of healthy appearance.

In the brain nothing remarkable beyond the fluidity and dark colour of the blood. On the third day after death, the spots could with difficulty be defined; their purple tint and that of the skin is changed to pink. A corresponding change had taken place upon a portion of skin which I had brought away to examine through the microscope.

Upon examining the spots on the mucous surface through a very powerful magnifying lens, they appeared to consist of a congeries of very loaded vessels with inconsiderable surrounding extravasation, and, on making a section of the spots on the skin, they presented to the unaided eye a similar arrangement.

My colleague, Mr. Barker, assisted me during the operation, and Dr. Marshall Hall, the physician to the Charity, was present at the post-mortem examination.

